# Evolution of Complex Thallus Alga: Genome Sequencing of *Saccharina japonica*

**DOI:** 10.3389/fgene.2019.00378

**Published:** 2019-05-02

**Authors:** Tao Liu, Xumin Wang, Guoliang Wang, Shangang Jia, Guiming Liu, Guangle Shan, Shan Chi, Jing Zhang, Yahui Yu, Ting Xue, Jun Yu

**Affiliations:** ^1^College of Marine Life Science, Ocean University of China, Qingdao, China; ^2^College of Life Sciences, Yantai University, Yantai, China; ^3^CAS Key Laboratory of Genome Sciences and Information, Beijing Key Laboratory of Genome and Precision Medicine Technologies, Beijing Institute of Genomics, Chinese Academy of Sciences, Beijing, China; ^4^University of Chinese Academy of Sciences, Beijing, China; ^5^College of Grassland Science and Technology, China Agricultural University, Beijing, China; ^6^Beijing Agro-Biotechnology Research Center, Beijing Academy of Agriculture and Forestry Sciences, Beijing, China; ^7^Qingdao Haida Blue Tek Biotechnology Co., Ltd, Qingdao, China; ^8^College of Biological Engineering, Qilu University of Technology, Shandong Academy of Sciences, Jinan, China; ^9^The Public Service Platform for Industrialization Development Technology of Marine Biological Medicine and Product of State Oceanic Administration, College of Life Sciences, Fujian Normal University, Fuzhou, China

**Keywords:** *Saccharina japonica*, genome sequencing, virus genome, phylogenetic analysis, extracellular components, halogen biosynthesis

## Abstract

*Saccharina*, as one of the most important brown algae (Phaeophyceae) with multicellular thallus, has a very remarkable evolutionary history, and globally accounts for most of the economic marine aquaculture production worldwide. Here, we present the 580.5 million base pairs of genome sequence of *Saccharina japonica*, whose current assembly contains 35,725 protein-coding genes. In a comparative analysis with *Ectocarpus siliculosus*, the integrated virus sequence suggested the genome evolutionary footprints, which derived from their co-ancestry and experienced genomic arrangements. Furthermore, the gene expansion was found to be an important strategy for functional evolution, especially with regard to extracelluar components, stress-related genes, and vanadium-dependent haloperoxidases, and we proposed a hypothesis that gene duplication events were the main driving force for the evolution history from multicellular filamentous algae to thallus algae. The sequenced *Saccharina* genome paves the way for further molecular studies and is useful for genome-assisted breeding of *S. japonica* and other related algae species.

## Introduction

Brown algae are a large group of multicellular algae, which displays a huge biomass dominating cool temperate intertidal and subtidal zone water, due to its macro soma and large biomes. Brown algae are fundamentally different from green and red algae, as green and red algae acquired plastids from cyanobacteria during primary endosymbiosis, while brown algae descend from secondary endosymbiosis (Valentin and Zetsche, 1990). Therefore, brown algae may acquire both cyanobacterial genes via EGT (Endosymbiotic Gene Transfer) and eukaryotic sequences from the nucleus of the red algal endosymbiont (Lane and Archibald, 2008), which makes the genome more complicated to interpret. Therefore, knowledge about the brown algal genome is crucial for understanding its evolution path. There are currently approximately 1500–2000 species of brown algae worldwide. The morphology of brown algae ranges from slender filaments (e.g., *Ectocarpus siliculosus*) to giant thallus (e.g., *Saccharina japonica*). Meanwhile, brown algae exhibit a diverse range of life cycles, for example the haploid–diploid cycle of the genus *Saccharina* is quite different from its close relative the genus *Ectocarpus* which lacks the parenchyma stage. This may have indicated key adaptive events in their evolution. One important question is which genes or evolutionary events underlie the structural evolution from filamentous brown algae (*Ectocarpus*) to heteromorphic haploid-diploid algae (*Saccharina*).

In addition, genus *Saccharina*, one of the most important genera of brown algae, has been recently separated from genus *Laminaria* based on molecular evidence from nuclear, plastid, and mitochondrial genome sequences (Draisma et al., 2001; Yoon et al., 2001; Erting et al., 2004; Lane et al., 2006). Many species from *Saccharina* are known to constitute the marine forests in the Asian coastal areas, which are the primary producers in marine ecosystem and traditionally indispensable in a diet with high industrial value (Zemke-White and Ohno, 1999). They are a potential source of renewable energy (Demirbas, 2010), as well as of polysaccharides, e.g., laminarans, alginic acids, and fucoidans (Vishchuk et al., 2011). *S. japonica* is not only a common seafood in China and many other countries, but has also been documented as a drug in traditional Chinese medicine for over a thousand years, being rich in polysaccharides, e.g., alginate, fucoidin, fucoidan galactosan sulfate and alginic acids, mannitol and trace elements. With the development of modern science and technology, the medical applications of *S. japonica* have been gradually revealed, such as its capacity to regulate blood lipids, blood sugar, and blood pressure, and its activities such as anticoagulation, antioxidant, anti-tumor and anti-radiation, etc. (Xue et al., 2001; Zhao et al., 2004, 2012; Kim et al., 2006; Wang et al., 2008, 2012; Huang et al., 2010; Mizuta and Yasui, 2010; Vishchuk et al., 2012).

A previous study on the transcriptome of *S. japonica* has facilitated the understanding of the genome background of *S. japonica* along the detailing of vanadium-dependent haloperoxidase family (Liang et al., 2014). Previous studies have shown the biosynthesis pathways of important cellular components (alginate and fucoidan) (Chi et al., 2018a) and complex halogen metabolism of *Saccharina* (Ye et al., 2015). The previous genome sequencing in *S. japonica* assembled 13,327 scaffolds (537-Mb) which covered 98.5% of the estimated genome and predicted 18,733 protein-coding genes, consisting of 13,327 scaffolds (Ye et al., 2015). It focused on the evolutionary adaptation and the functional diversification of the polysaccharide biosynthesis and iodine concentration mechanisms of *S. japonica*. However, a better genome assembly is necessary to provide a complete understanding of *S. japonica*, and further reveal the evolution significance of multicellular parenchymatous thallus. In particular, the Hi-C (High-through chromosome conformation capture) data can be used for clustering the scaffolds into chromosomes, by exploiting chromatin interaction data (Korbel and Lee, 2013). A complete genome assembly of *S. japonica* will further facilitate our research with the evolution pattern of tissue differentiation and heteromorphic generational alternation. Comparative genomics analysis will give us a deeper insight into the gene family duplication, differential expansion/contraction and evolution of conserved non-coding sequences. Meanwhile, a detailed investigation of *S. japonica* genome will accelerate the metabolic expatiation of mannitol, laminarin, alginates and trehalose pathways.

Here, we surveyed the genome of *S. japonica* with the help of high throughput sequencing. We intend to include a detailed analysis of the updated genome data of *S. japonica*, which will pave the way of its genetic research and breeding. We reported a preliminary analysis of its genome organization and gene content, including gene annotations, GC content, repeat elements and SSRs. Subsequently, phylogenetic analysis, motif analysis and exon-intron organization of some important genes were also investigated. We believe the *S. japonica* genome generated in this study will enhance both fundamental and applied research in related areas.

## Materials and Methods

### Sample Collection and DNA Preparation

A thermotolerant and high-yielding *Saccharina* cultivar “Rongfu” was selected as the genomic DNA source for whole genome sequencing. Samples were collected from Rongcheng, Shandong Province, P. R. China, and were provided by the Culture Collection of Seaweed at the Ocean University of China. Genomic DNA was extracted from fresh sporophyte with improved CTAB method (Guillemaut and Maréchal-Drouard, 1992).

### Genome Sequencing and Assembly

We constructed three paired-end libraries and three mate-paired libraries according to Illumina standard operating procedure. Sequencing of each library was performed on an Illumina HiSeq 2000 instrument to produce the raw data. We then filtered out low-quality and short reads to obtain a set of usable reads.

We then assembled the reads into contigs using SOAPdenovo with varying parameters, and mate-paired relationships between the reads were used to construct scaffolds. The genome assembly was improved by exploiting chromatin interaction data from Hi-C data, and grouping contigs into pseudomolecules/chromosomes. Chromatins were cut by the restriction enzyme *Mbol*, and ligated together *in situ* after biotinylation. DNA was extracted, and sheared before end repairing. DNA fragments were enriched by using interaction of biotin and streptavidin, and subject to Hiseq sequencing with a paired end length of 150 bp. After trimming and quality control, paired-end reads were aligned to the genome assembly, and the reads with more than one hit were discarded. The reads without the restriction site of *Mbol* were filtered out. Then, paired-end reads were analyzed for the valid ones, which were aligned to two different enzyme fragments, for the estimated insert size to meet the expectations, by using HiC-Pro v2.7.8 (Servant et al., 2015). Based on the relationships among valid reads, the order and directions of scaffolds/contigs were clustered into the 31 pseudomolecules/chromosomes by LACHESIS (Burton et al., 2013).

### Repeat Analysis and Genome Annotation

Both homology-based and *de novo* prediction analyses were used to identify the repeat content in the *Saccharina* genome. For the homology-based analysis, we used Repbase (version 20140131) to perform a TE search with RepeatMasker (open-4.0) and the ncbi RMblast search engine. For the *de novo* prediction analysis, we used RepeatModeler to construct a TE library, and classified elements within the library using a homologous search with Repbase and TEClass.

Approaches including homology detection, expression-evidence-based predictions and *ab initio* gene predictions were used for gene model construction. To identify homology patterns in *S. japonica*, the BLASTX search was performed against the NCBI non-redundant protein database with *E*-value < 10^-5^. For expression evidences, published ESTs, transcripts and RNAseq datasets from the OneKP database^1^ were aligned to the genome. After measuring and comparing a variety of programs, AUGUSTUS was used for *ab initio* gene prediction. Gene model parameters for the programs were trained from long transcripts and known *Saccharina* genes processed by PASA. And then all these predictions were combined into consensus gene structures using EVM and optimized by manual corrections.

Functional classification of Gene Ontology of the genes was performed with InterProScan (Zdobnov and Apweiler, 2001). The EuKaryotic Orthologous Groups (KOG) classification was performed against KOG database (Tatusov et al., 2003). Pathway analyses were performed using the Kyoto Encyclopedia of Genes and Genomes (KEGG) annotation service KAAS (Kanehisa et al., 2004).

### Gene Family Analysis

Related protein sequences were downloaded from NCBI and local BLAST was used to get full-length sequences in *S. japonica*, with tBLASTn module of WU-BLAST 2.0 and an *E*-value cut-off of 1 × 10^-5^. Nucleotide sequences were transferred to amino acid sequences using MEGA with option of standard genetic code and then aligned (Tamura et al., 2011). NCBI BLAST was run on proteins sequences sets of *S. japonica*, *E. siliculosus*, *Aureococcus anophagefferens*, *Nannochloropsis gaditana*, *Phaeodactylum tricornutum*, and *Thalassiosira pseudonana*, which were downloaded from the NCBI GenBank, and then were clustered to orthogroups using Orthomcl (Li et al., 2003). The *S. japonica* assembly was compared to the available *E. siliculosus* genome^2^ using lastz (Frith et al., 2010) with – chain and – gapped parameter, and then the alignments were plotted for synteny analysis.

### Phylogeny Analysis

In phylogenetic analysis, full amino acid sequences of housekeeping genes from archaea, proteobacteria, cyanobacteria, tracheophytes, and algae, were aligned using MEGA6 (Tamura et al., 2013) software and edited manually. MrBayes v3.1.2 (Ronquist and Huelsenbeck, 2003) software was used to investigate evolutionary relationships based on amino acid sequences. Bayesian analysis was performed by two separate sequence analyses for four Markov chains (using default heating values), which were run for 500,000 generations until the average standard deviation of split frequencies was below 0.01 (Posada and Crandall, 1998). In addition, trees were sampled every 100 generations with the first 25% of trees discarded as the burn-in. Remaining trees were used to build a 50% majority rule consensus tree, accompanied with posterior probability values. FigTree v1.3.1^3^ was used for displaying phylogenetic trees.

## Results and Discussion

### Genome Assembly

The genome sequence was assembled using 14.86 and 15.06 Gb mate-paired sequences from libraries with 3 and 5 kb inserts, respectively, plus 46.54 Gb paired-end reads from small-insert libraries (Supplementary Table S1). The assembled nuclear genome of *S. japonica* contains 418,683 contigs and 236,802 scaffolds, and the total length was 580.5 Mb (Table 1 and Supplementary Table S2), with an N50 of 13,636,083 bp and 48.72% GC content. There are 1,602 scaffolds longer than 100 kb, a total of 5,257 longer than 20 kb, and a total of 11,156 that are longer than 10 kb. We further improved the assembly by using the Hi-C data. After trimming, a total of 237,795,214 pair-end reads, and 105,783,030 pair-end reads were determined as valid, which were aligned to two different enzyme fragments, and the estimated insert size met the expectation. Based on the relationships among valid reads, the order and directions of 46,865 scaffolds/contigs were clustered into the 31 pseudomolecules/chromosomes (Supplementary Table S3), which account for 517,689,860 bp, about 89.19% of the whole genome. This was an updated genome assembly and provided a better resource than the previous assembly (Ye et al., 2015).

**Table 1:** Genome statistics of *S. japonica* and *E. siliculosus*.

	*S. japonica*	*E. siliculosus*
Genome size (Mb)	580.5	195.8
Number of scaffolds > 2 kb	11,156	1561
Supercontig (scaffold) N50 (bp)	123,490	504,428
Number of contigs	418,683	14,403
Contig N50 (bp)	4733	32,862
% of cDNAs matching the genome	91.28%	97.40%
G+C content	48.72%	53.60%
% of repeated sequences	46.03%	22.70%
Number of genes	35,725	16,256
Average gene length (bp)	5591	6859
Average coding sequence length (bp)	1152	1563
Number of introns	131,519	113,619
Average intron length (bp)	1203	703.8
Average number of introns per gene	4.63	6.98
Number of exons	167,244	129,875
Average exon length (bp)	250.27	242.2
Number of single exon genes	7051	856
Number of genes (Blast *e*-value cutoff, e^-10^)	27,251(76.3%)	10,278 (63.2%)
Number of genes with expressed sequence support	30,595 (85.64%)	9601 (59%)

A combination of expert and automatic annotation predicts 35,725 gene models, with an overlap of 23,930 ones with *E. siliculosus* (Supplementary Figure S1). Genes are rich in introns (4.63 per gene on average). However, the analysis of GO annotation, KOG and KEGG discovered a similar distribution between *S. japonica* and *E. siliculosus* (Supplementary Figures S2–S4). A gene family clustering analysis across the whole genome was conducted by using protein sequences of *S. japonica* and *E. siliculosus*. Totally, there were 16,954 genes in 9658 orthogroups in *S. japonica*, 13,063 genes in 9615 orthogroups in *E. siliculosus*, with 18,771 and 3471 genes unassigned, respectively (Table 2). We extended our ortholog search to more species, i.e., *A. anophagefferens*, *N. gaditana*, *P. tricornutum*, and *T. pseudonana*, and found that the gene number in orthogroups of *S. japonica* is the highest, suggesting that the gene duplication strategy was preferred during the genome evolution (Table 2).

**Table 2:** Orthologous gene number in six algal species.

	*Saccharina japonica*	*Ectocarpus siliculosus*	*Nannochloropsis gaditana*	*Phaeodactylum tricornutum*	*Thalassiosira pseudonana*	*Aureococcus anophagefferens*
Gene number	35,725	16,534	9053	10,402	11,776	11,501
Gene number in orthogroups	16,954	13,063	6460	8316	10,127	7832
Number of unassigned genes	18,771	3471	2593	2086	1649	3669
Number of orthogroups containing species	9658	9615	5229	6237	7598	4531
Number of species–specific orthogroups	66	17	4	20	16	50
Gene number in species–specific orthogroups	435	61	8	109	69	191

### Integration of Virus Genome in Co-ancestry Period of Laminariales and Ectocarpales

**FIGURE 1:**
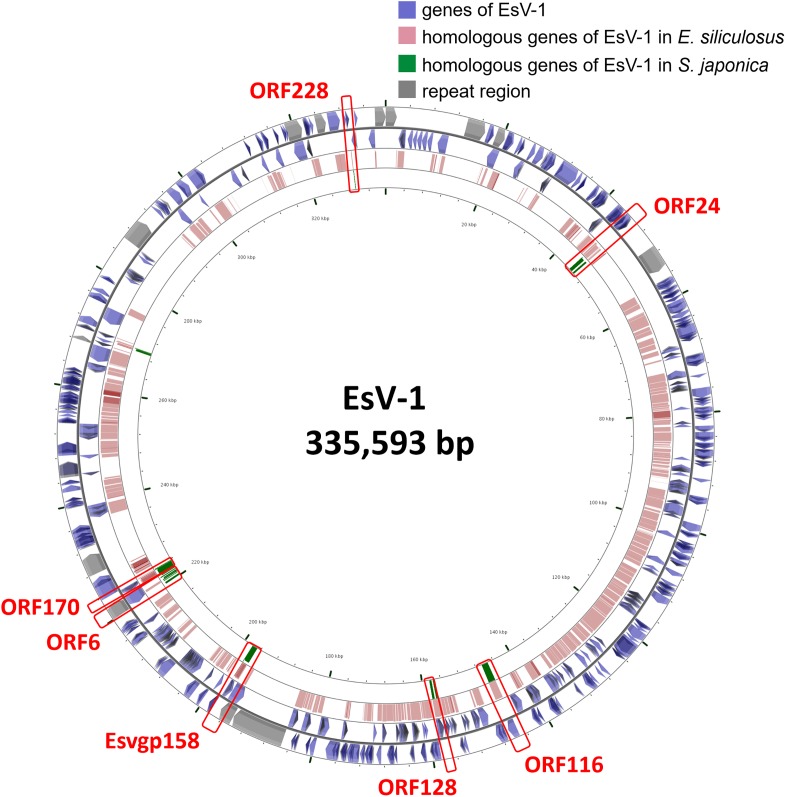
The viral EsV-1 genome and its fragments integrated in the *S. japonica* and *E. siliculosus* genomes. The genes highlighted in red bar are found in both *S. japonica* and *E. siliculosus* genomes.

Laminariales and Ectocarpales algae show a close phylogenetic relationship with each other. Analysis of the *E. siliculosus* genome sequence identified the presence of *E. siliculosus* virus-1 (EsV-1) (Cock et al., 2010). EsV-1 only infects free-swimming gametes or spores. The viral DNA integrates into the cellular genome after infection and then, through mitosis, it spreads to all cells of a developing host (Bräutigam et al., 1995; Lee et al., 1998). Viral DNA has been detected in some species’ genomes in *Ectocarpus* and *Feldmannia* (Meints et al., 2008; Cock et al., 2010). In our analysis, we found some genomic fragments of phaeovirus EsV-1 integrated in *S. japonica* genome, which were also found in *E. siliculosus* genome (Figure 1). This result shows that this virus integration may happen before the divergency of Laminariales and Ectocarpales. After BLAST against EsV-1 sequences, we also found that the integrated virus sequences are quite different in these two genomes, as *E. siliculosus* genome contains large-fragment clusters (310,438 bp of 313,838 bp EsV-1 genome, 98.92%, and contains 173 orthologs genes of 231 EsV-1 ones), and in *S. japonica* the orthologous regions consist of more fragments and fewer large segments (8,256 bp). So the integration would have occurred before the differentiation of Laminariales and Ectocarpales, and experienced the isolated evolution events after the differentiation. This indicates the diverse algal genome evolutionary mechanisms between Laminariales and Ectocarpales, which may represent the potential driving force for advanced brown algae evolutionary. There may be large-scale rearrangement in Laminariales genomes during evolution from filaments to the thallus, accompanied by loss of some genes.

### Repeats, Introns and Non-coding Regions

We downloaded the genome sequences of brown algae species *E. siliculosus* (Michel et al., 2010), and conducted a synteny analysis. In total, the conserved regions covered 23,193,396 bases from *S. japonica* and 15,108,600 bases from *E. siliculosus*, and no significant replication of large fragments were shown (Supplementary Figure S5). We found that 43.12% of the *S. japonica* genome assembly could be attributed to repeat sequences, while 22.7% only was found for the reported 214 Mb genome of *E. siliculosus*, a multicellular filamentous brown alga. LTR, LINE, SINE and simple repeat are the major components of repeat sequences (Supplementary Figure S6).

In total, 98,386 SSRs conforming to the definitions (i.e., unit/minimum number of repeats 1/20, 2/10, 3/7, 4/5, 5/4, and 6/4) were recovered from 15,173 sequences (6.28% of the total sequences) in the *S. japonica* genome sequences. Provided that the size of the *S. japonica* genome is 580.5 Mb, the frequency of occurrence of the above SSRs was estimated to be one SSR per 59 kb. Compound SSRs accounted for 17.75% and the average SSR density was 169 SSR/Mb. Di-, tri-, and tetra-nucleotide SSRs accounted for 4.51, 46.67, and 17.21% of the identified SSRs, respectively.

When it comes to the di-nucleotide SSRs, AG/CT pattern was the most frequent type representing 41.06% of di-nucleotide repeat units, followed by AT/AT (35.74%), and AC/GT (23.09%) (Supplementary Figure S7). AGC/CTG was the most abundant trimer motif (34.11%), and ACT/AGT was second at 23.77%. Among the tetra-nucleotide SSRs, ACAG/ATGT (18.20%) was the most common and ACCCG/CGGGT (13.57%) was the most common hexamer (5.00%).

Genome analysis of *S. japonica* predicted 35,725 genes with average intron length of 1,203 bp, 4.63 introns per gene. Introns constitute 29% of the whole genome, which is lower than that of *E. siliculosus* (37.3%). A total of 15,820 simple sequence repeats (SSRs) were identified. Among the SSRs, the trinucleotide and dinucleotide repeat types are the most abundant, which is similar to other published algae genome data.

### Housekeeping Genes

**FIGURE 2:**
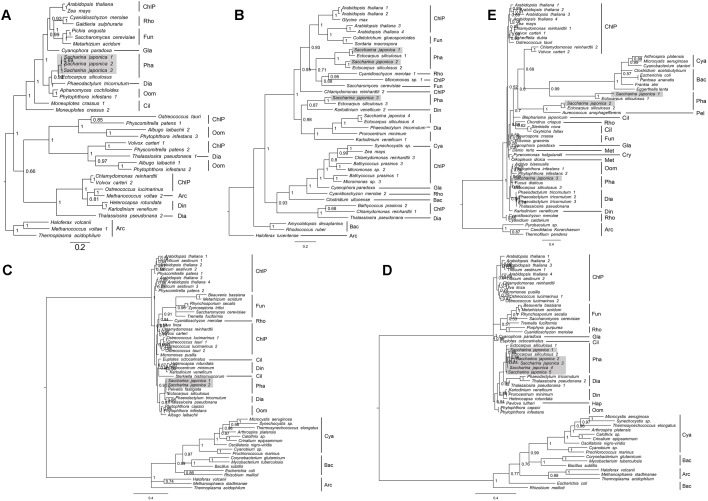
Phylogenetic tree topologies of housekeeping genes using MrBayes software. **(A)** EF1α, **(B)** GAPDH, **(C)** Actin, **(D)** TUA, and **(E)** TUB. ChlP, Chlorophyta; Rho, Rhodophyta; Fun, Fungi; Pha, Phaeophyceae; Dia, Diatom; Oom, Oomycetes; Cya, Cyanobacteria; Bac, Bacteria; Arc, Archaea; Hap, Haptophyta; Cry, Cryptophyta; Gla, Glaucophyta.

Just like the diverse archaea, bacteria, tracheophytes and algal species, a wealth of candidate housekeeping gene sequences, such as *actin*, α-*Tubulin* (*TUA*), ß-*Tubulin* (*TUB*), Elongation factor 1-alpha (*EF1-*α), and glyceraldehyde 3-phosphate dehydrogenase (*GAPDH*) were also detected in the genome of *S. japonica*. Therefore, we built phylogenetic trees that display relationships of full amino acid sequences of housekeeping genes from archaea, proteobacteria, cyanobacteria, tracheophytes, and algae (only representative candidates are included to save space) based on Bayesian method. In the consensus tree of four housekeeping genes *(EF1α*, *GAPDH*, *TUA*, and *TUB*), almost all the Phaeophyceae algae (including *S. japonica*) formed a well-supported clade with oomycetes or protists, which indicates their origin from endosymbiosis host (Figure 2). The remaining *actin* genes from brown algae have a complex evolutionary history. Our phylogenetic analysis shows they may have arisen from multiple origins. Three copies of *S. japonica actin* cluster into separate clades: one is related to oomycetes (e.g., *S. japonica* 1), and the other groups with cyanobacteria and bacteria (e.g., *S. japonica* 2, 3). Therefore, *S. japonica* may acquire *actin* from different ancestors, while *S. japonica* 1 is with an endosymbiotic host origin, and *S. japonica* 2 and 3 have a cyanobacterial origin through endosymbiosis gene transfer (EGT) or acquired from non-cyanobacterial proteobacteria via horizontal gene transfer (HGT).

### Gene Expansion

During the evolution of single cells to multicellular, housekeeping genes, signal transduction and cell junction pathway related genes play an important role (Supplementary Figures S2, S3), while genes and pathways of extracellular component may be more important from multicellular filamentous to multicellular thallus, due to gene expansion (Table 3).

**Table 3:** Gene number of different metabolisms and gene families in sequenced Heterokontophyta algae.

	*Saccharina japonica*	*Ectocarpus siliculosus*	*Nannochloropsis gaditana*	*Phaeodactylum tricornutum*	*Thalassiosira pseudonana*	*Aureococcus anophagefferens*
Cellulose synthase	14	9	3	–	–	-
Glycosyltransferase	194	88	11	62	78	129
Glycosyl hydrolase	107	41	14	30	55	85
Sulfotransferase	8	13	5	24	34	38
Mannitol synthesis	6	7	2	1	–	2
Alginate synthesis (except GTs)	96	34	2	3	2	1
Mannuronate C5-epimerase	84	26	–	–	–	–
Fucose synthesis (except GTs and STs)	5	5	2	4	3	4
Protein kinase	369	258	55	84	132	258

In phylum Heterokontophyta, there is a very close relationship between orders Laminariales and Ectocarpales, which is consistent with phylogenetic analysis of chloroplast genome. In the present study, in *S. japonica* the genome size is almost 3 times that of *E. siliculosus*, and the total gene number is nearly 2.1 times that of *E. siliculosus* (Tables 2, 3). *S. japonica* and *E. siliculosus* genes were subjected to the KEGG database, and the annotated gene comparison revealed a similar distribution of gene types among most categories, which indicated the increases in organismal complexity are not associated with pathway changes. The similarity of gene orthologous were also compared, which suggests that the gigantic increasing of *S. japonica* genes may depend on gene replication leading to multiple copies. Interestingly, analysis of gene duplication indicated a significant gain of the ones associated with cell wall component metabolisms such as alginate and cellulose synthesis pathways. For instance, *Saccharina* genome contains a number of 14 candidate cellulose synthase and cellulose synthase-like genes, which is nearly 1.6 times that of *Ecutocarpus* (Table 3). The modifier gene *MC5E* in alginate synthesis pathway reached 84 copies in *Saccharina* genome, about 3.2 times that of *Ecutocarpus*. The gene number of glycosyltransferases (*GT*s) and glycosyl hydrolases (*GH*s), which are likely involved in cell wall polysaccharide metabolisms, also endures an expansion of more than two times in *Saccharina* genome. The same situation occurred in protein kinases. These genes include membrane-spanning receptor kinases, which may play key roles in developmental processes such as differentiation (De Smet et al., 2009). This indicated that the strategy of gene expansion may contribute a lot to the evolution from the multicellular filamentous brown alga to complex thallus alga.

*Saccharina japonica* is the most efficient iodine accumulator among all living organisms, owing to the activities of vanadium-dependent haloperoxidases (*vHPOs*) (Leblanc et al., 2006). The halogen accumulation level of foliaceous *S. japonica* is much higher than that of filamentous *E. siliculosus* and the halogen metabolism ability is significantly enhanced. In the genome of *S. japonica*, we identified 89 *vHPOs*, including 21 vanadium-dependent bromoperoxidases (*vBPO*s) and 68 vanadium-dependent iodoperoxidases (*vIPO*s). In contrast, the previous genomic study on *E. siliculosus* and *S. japonica* has, respectively, predicted one (Table 4) and 76 *vHPO* genes (Ye et al., 2015) involved in halogen metabolism. In addition, 32 dehalogenation related genes were predicted. Brown algae usually have active halogen metabolism, and many halogen related genes were found in the *E. siliculosus* and *Chondrus crispus* genome (Saenko et al., 1978; Leblanc et al., 2006). Hypohalous acids and organo-halogenated compounds produced by halogenations are considered substances which can participate in algae defense reactions. The large size of these halogen-related gene families is a result of specific evolutionary adaptation to the marine environment, and it may be evolved in various defense mechanisms and produce more rich secondary metabolites in evolution from small algae to large algae.

**Table 4:** Gene copies in the important gene pathways of *S. japonica* and *E. siliculosus*.

Pathway	Gene	*S. japonica*	*E. siliculosus*
Halogen biosynthesis	*vHPO*	89	1
Mannitol biosynthesis	*M1PDH*	2	3
	*M1Pase*	2	2
Alginate and fucoidan biosynthesis	*MPI*	3	4
	*PMM/PGM*	1	2
Alginate biosynthesis	*GMD*	2	3
	*UGD*	1	1
Fucoidan biosynthesis	*GM46D*	2	2
	*GFS*	1	1

Plants respond to various biotic or abiotic stimuli with different mechanisms. Heat shock proteins (*Hsps*) are essential components in plant tolerance mechanism under various abiotic stresses. The function of calcium-based intracellular signaling system is to combine extracellular stimuli with their specific intracellular responses (Edel et al., 2017). Three major elements play a role in the generation and translation of a stimulus-induced Ca^2+^ signal: influx, efflux and decoding (Edel et al., 2017). In a comparison with *E. siliculosus*, *S. japonica* experienced a gene expansion in *Hsp* family. The number of *hsp* genes including *Hsp*20, *Hsp*33, *Hsp*40, *Hsp*70, and *Hsp*90 in *S. japonica* is nearly 1.5 times of that of *E. siliculosus* (Table 5). In the genome of *S. japonica*, we identified 3 protein families of Ca^2+^ influx, 2 protein families of Ca^2+^ efflux and 2 protein families of calcium decoding (Table 6). The number of *TPC*, *MCA*, and *CaM* genes were higher than in *E. siliculosus* (Table 6). These gene expansion events may contribute to the differentiation and evolution from filamentous *Ectocarpus* to complex organized *Saccharina*. In addition, we also found more gene copies of *Hsp*40, *Hsp*70, *TPC*, *MCA*, and *CPK* in our *S. japonica* “Rongfu” genome than in the previous *S. japonica* genome (Ye et al., 2015). It indicated that the *Saccharina* high-temperature-resistant variety “Rongfu” possessed more genes of *Hsp* and calcium-based signaling system which might be caused by artificial selection.

**Table 5:** Gene number of *Hsp* gene families in sequenced *Saccharina* variety “Rongfu,” *S. japonica* and *E. siliculosus*.

	*Hsp* 20	*Hsp* 33	*Hsp* 40	*Hsp* 70	*Hsp* 90	Total
*Saccharina* variety “*Rongfu*”	1	2	12	20	7	42
*S. japonica*	1	2	8	17	7	35
*E. siliculosus*	1	1	8	11	4	25

**Table 6:** Gene number of the calcium-based signaling system in sequenced Saccharina variety “Rongfu,” *S. japonica* and *E. siliculosus*.

	Ca^2+^ Influx system			Ca^2+^ Efflux system		Calcium decoding system		Total
	*TPCs*	*MCAs*	*VDCCs*	*ACAs*	*HAM1*	*CaMs*	*CPKs*	
*Saccharina* variety “*Rongfu*”	5	5	1	2	3	5	21	41
*S. japonica*	3	4	1	2	3	5	18	36
*E. siliculosus*	3	4	1	2	3	3	21	37

The above results indicated that the expansion of *Saccharina* genome was mainly due to gene family expansion, especially when it contributed to cell wall and halogen biosynthesis (Tables 3–6). The differentiation and evolution from filamentous *Ectocarpus* to complex organized *Saccharina* may be related to these gene expansion events.

However, we did not discover a huge replication in housekeeping, signal-transduction-related and cell-communication-related genes. For example, mannitol represents up to 15–26% of dry weight, as one of the primary photosynthetic products and storage compounds in Laminariales, and its biosynthesis involves two major enzymes, mannitol-1-P dehydrogenase (*M1PDH*) and mannitol-1-phosphatase (M1Pase) (Chi et al., 2018b). Two unigenes of *M1PDH1* and *M1PDH2* and two *M1Pase* homologs of *M1Pase1* and *M1Pase2* were found in the *S. japonica* genome, while there are three *M1PDH* unigenes and two *M1Pase* copies in *E. siliculosus* (Table 4). Meanwhile, in a comparison of *E. siliculosus*, *S. japonica* did not experience a significant family expansion in alginate and fucoidan biosynthesis, which involves 6–8 genes/families (Chi et al., 2018a). Mannose-6-phosphate isomerase (*MPI*), phosphomannomutase (*PMM*), and mannose-1-phosphate guanylyltransferase (*MPG*) are involved in converting fructose-6-phosphate into GDP-mannose. GDP-mannose may be used in the two biosynthesis ways: the alginate biosynthesis which involves GDP-mannose/UDP-glucose 6-dehydrogenase (*GMD/UGD*), mannuronan synthase (*MS*), and *MC5E*; the fucoidan biosynthesis which involves GDP-mannose 4,6-dehydratase (*GM46D*), GDP-fucose synthetase (*GFS*), and *et al*. It showed that *S. japonica* contained less gene copies of *MPI*, *PMM/PGM*, and *GMD*, compared to *E. siliculosus* (Table 4).

In addition, we also found more gene copies in our *S. japonica* than in the previous *S. japonica* genomes (Ye et al., 2015), for example, the *vHPO* gene family. It indicated that the *Saccharina* varieties possess the different gene clusters, which might be caused by artificial selection, and provide important resources for algal breeding on high alginate and iodine.

### Data Availability

The raw data is deposited in NCBI, with Bioproject accession of PRJNA280905, and the accession in Short Read Archive of SRP057092, including four running datasets, SRR1972526, SRR1972528, SRR1972529, and SRR1972530.

## Conclusion

Large alga *S. japonica* has a large complex thallus tissue, and possesses a huge genome and gene expansion. In spite of high similarity in gene composition and classification, the structures of the two genomes of *E. siliculosus* and *S. japonica* are different in non-coding regions, repeat sequences, introns length and gene number. In particular, the number of genes related to extracellular components and halogen biosynthesis in *S. japonica* is significantly higher than that of *E. siliculosus*, which may be the main motive force for evolution of filament to thallus. In addition, the integration of viral genome in the *S. japonica* and *E. siliculosus* genomes during their co-ancestry period further demonstrated their close genetic relationship, genome rearrangements and gene duplication events after their differentiation.

## Author Contributions

TL, TX, XW, and JY designed the study. SC, JZ, and YY maintained and prepared the plant materials. GL and GS prepared the sequencing libraries and conducted the sequencing. TX conducted the Hi-C sequencing. GW, SJ, and XW assembled the draft sequence, and conducted the analysis. GW and SC drafted the manuscript. SJ modified the manuscript. All authors reviewed and approved the final manuscript.

## Conflict of Interest Statement

SC was employed by Qingdao Haida Blue Tek Biotechnology Co., Ltd. The remaining authors declare that the research was conducted in the absence of any commercial or financial relationships that could be construed as a potential conflict of interest.

## Footnotes

1 https://db.cngb.org/onekp/

2 http://bioinformatics.psb.ugent.be/genomes/view/Ectocarpus-siliculosus

3 http://tree.bio.ed.ac.uk/software/figtree/
