# Supplementary material for: Evolution of Complex Thallus Alga: Genome Sequencing of Saccharina japonica
Source: Front Genet. 2019 May 2;10:378. doi: 10.3389/fgene.2019.00378 (PMC6507550; doi:10.3389/fgene.2019.00378)
Supplement: Supplementary file 1 [file Table_1.DOCX]

| **TABLE S1 \|** Raw data from Hiseq sequencing | | |  |  |  |
| --- | --- | --- | --- | --- | --- |
| **Libraries (insert size)** | **Platform** | **Raw Data (Gb)** | **Read length (bp)** | **Number of Reads** | **Coverage** |
| Paired-end (180 bp) | Illumina | 8.82 | 111 | 42,670,556 | 16.17 |
| Paired-end (500 bp) | Illumina | 19.41 | 121 | 86,104,513 | 35.56 |
| Paired-end (800 bp) | Illumina | 18.31 | 111 | 88,569,356 | 33.56 |
| Mate-paired (3 kb) | Illumina | 14.86 | 101 | 78,986,750 | 27.23 |
| Mate-paired (5 kb) | Illumina | 15.06 | 101 | 80,028,864 | 27.59 |
| Total |  | 76.46 |  |  | 140.11 |

| **TABLE S2 \|** Assembly summary by Hi-C | | |  |  |
| --- | --- | --- | --- | --- |
| **Items** | **Contig_len(bp)** | **Contig_num** | **Scaffold_len(bp)** | **Scaffold_num** |
| Total | 525,625,349 | 418,683 | 580,992,664 | 236,802 |
| Max | 137,121 | - | 69,755,864 | - |
| Number>=2000bp | - | 67,064 | - | 6,185 |
| N50 | 4,741 | 26,920 | 13,636,083 | 15 |
| N60 | 3,426 | 39,992 | 13,187,666 | 19 |
| N70 | 2,341 | 58,527 | 12,464,467 | 24 |
| N80 | 1,420 | 87,235 | 5,440,044 | 29 |
| N90 | 608 | 142,212 | 4,609 | 3,696 |

| **TABLE S3 \|** Chromosome size and scaffold number | | |
| --- | --- | --- |
| **Pseudomolecule** | **Scaffold Num** | **Length** |
| chr1 | 603 | 69,755,864 |
| chr2 | 198 | 21,346,006 |
| chr3 | 230 | 19,031,613 |
| chr4 | 189 | 18,396,962 |
| chr5 | 181 | 18,153,460 |
| chr6 | 178 | 17,449,648 |
| chr7 | 161 | 16,782,660 |
| chr8 | 173 | 16,266,826 |
| chr9 | 181 | 15,673,945 |
| chr10 | 166 | 15,019,338 |
| chr11 | 166 | 14,070,410 |
| chr12 | 164 | 14,183,722 |
| chr13 | 165 | 14,089,445 |
| chr14 | 144 | 14,069,662 |
| chr15 | 154 | 13,187,666 |
| chr16 | 141 | 13,636,083 |
| chr17 | 153 | 13,179,656 |
| chr18 | 140 | 12,959,013 |
| chr19 | 142 | 13,491,692 |
| chr20 | 131 | 13,246,626 |
| chr21 | 143 | 13,493,318 |
| chr22 | 137 | 12,839,137 |
| chr23 | 138 | 12,680,716 |
| chr24 | 117 | 12,464,467 |
| chr25 | 120 | 11,921,893 |
| chr26 | 128 | 11,047,583 |
| chr27 | 105 | 11,355,836 |
| chr28 | 106 | 10,627,074 |
| chr29 | 83 | 5,440,044 |
| chr30 | 78 | 5,392,672 |
| chr31 | 89 | 4,296,616 |
| Total anchored | 5004 | 475549653 |
| Unanchored | 236,771 | 105,443,011 |
