# Supplementary material for: Evolution of Complex Thallus Alga: Genome Sequencing of Saccharina japonica
Source: Front Genet. 2019 May 2;10:378. doi: 10.3389/fgene.2019.00378 (PMC6507550; doi:10.3389/fgene.2019.00378)
Supplement: Supplementary file 2 [file Table_2.DOCX]

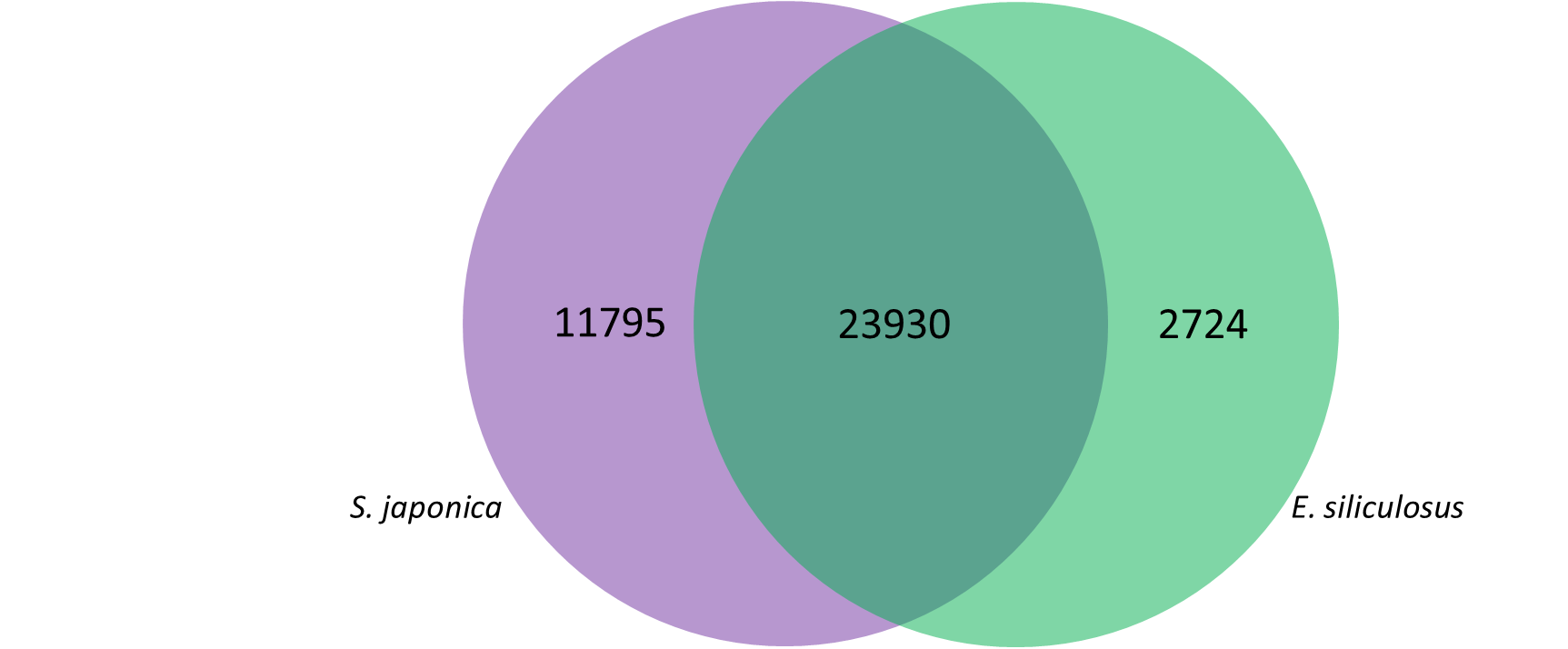


**FIGURE** **S1 |** Gene content compared with *E. siliculosus*.


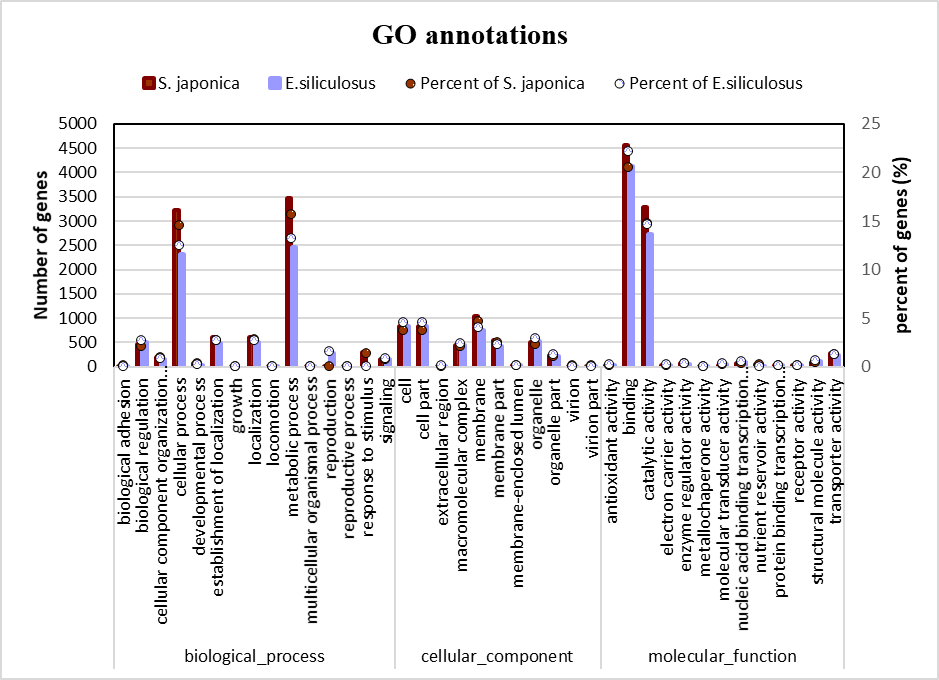


**FIGURE S2 |** GO annotations.


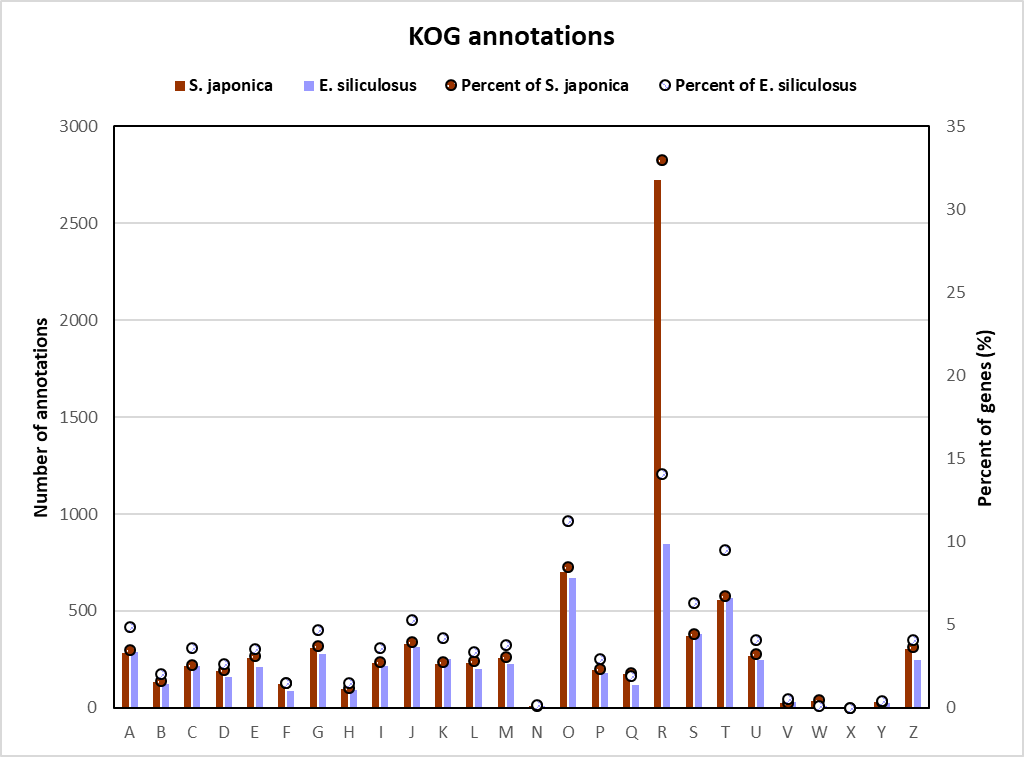


**FIGURE S3 |** KOG annotations.

One-letter functional classification used in the KOG was listed as follows,

INFORMATION STORAGE AND PROCESSING

[J] Translation, ribosomal structure and biogenesis

[A] RNA processing and modification

[K] Transcription

[L] Replication, recombination and repair

[B] Chromatin structure and dynamics

CELLULAR PROCESSES AND SIGNALING

[D] Cell cycle control, cell division, chromosome partitioning

[Y] Nuclear structure

[V] Defense mechanisms

[T] Signal transduction mechanisms

[M] Cell wall/membrane/envelope biogenesis

[N] Cell motility

[Z] Cytoskeleton

[W] Extracellular structures

[U] Intracellular trafficking, secretion, and vesicular transport

[O] Posttranslational modification, protein turnover, chaperones

METABOLISM

[C] Energy production and conversion

[G] Carbohydrate transport and metabolism

[E] Amino acid transport and metabolism

[F] Nucleotide transport and metabolism

[H] Coenzyme transport and metabolism

[I] Lipid transport and metabolism

[P] Inorganic ion transport and metabolism

[Q] Secondary metabolites biosynthesis, transport and catabolism

POORLY CHARACTERIZED

[R] General function prediction only

[S] Function unknown


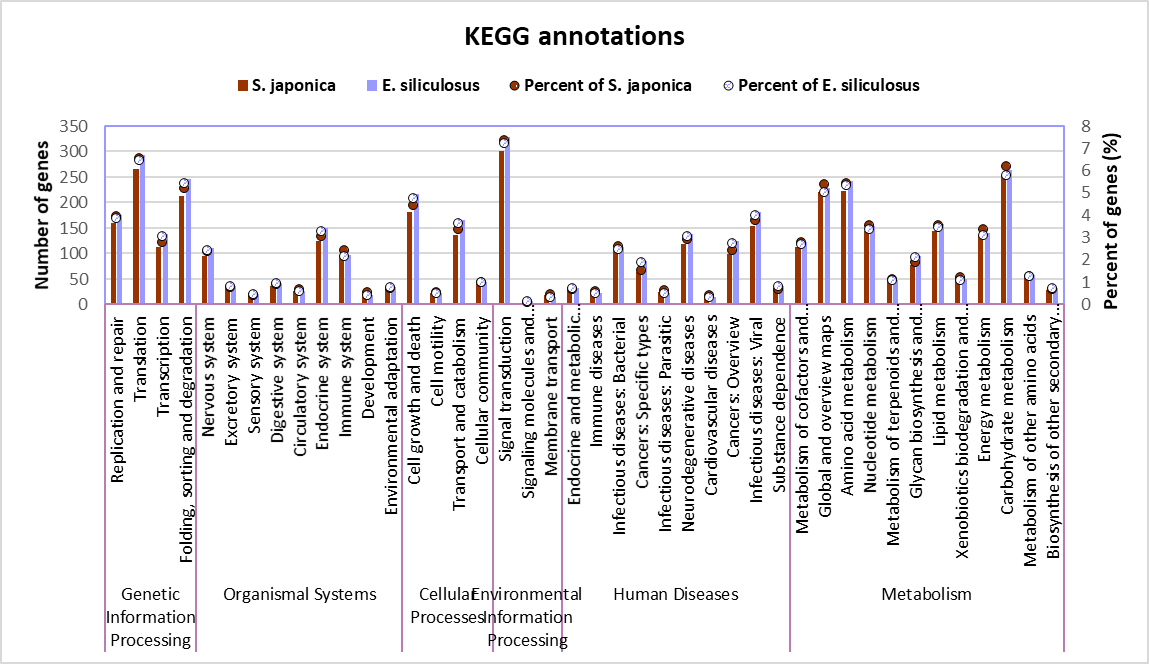
**FIGURE** **S4 |** KEGG pathway analysis.

**FIGURE S5 |** Genome-wide synteny analysis between *S. japonica* and *E. siliculosus*.

**FIGURE S6 |** Relative constituents of repeated sequences in *S. japonica* genome

**FIGURE** **S7 |** Distribution of SSR motifs.
